# Reducing Central Line Associated Bloodstream Infections via Improved Dressing Adherence in Patients with Coagulopathy

**DOI:** 10.1017/ash.2025.292

**Published:** 2025-09-24

**Authors:** Petra Ostrom

**Affiliations:** 1 Adventhealth Orlando

## Abstract

**Background:** Patients with cirrhosis often experience coagulopathy, which can result in profound bleeding at intravenous insertion sites. This makes maintaining dry, intact central venous catheter (CVC) dressings particularly challenging. At our 2,247-bed acute care tertiary referral hospital, the Medical-Surgical Intensive Care Unit (MSICU) specializes in hepatic care.

Upon completing a root cause analysis of our elevated central line associated blood stream infections (CLABSIs), we found patients with coagulopathy had poor CVC dressing adherence. Our goal was to reduce CLABSIs by improving dressing integrity through innovative strategies aimed at mitigation of bleeding and enhanced adhesion. **Method:** In November 2022 a review was completed of hemostatic and adhesion products to address bleeding at CVC insertion sites and improve dressing adherence to skin. In December 2022 we developed a tiered intervention program using three products tailored to the severity of bleeding at CVC insertion sites. We then selected an adhesive product to bond the perimeter of the dressing to the skin. We disseminated education to the nursing team on product use according to patient CVC dressing condition and manufacturer instructions for use. The tiered intervention program was evaluated by comparing pre-intervention (January 2021 to November 2022) CLABSI rates and standardized infection ratios (SIRs) to post-intervention (December 2022 to October 2024) outcomes. Data was obtained from the National Healthcare Safety Network (NHSN), and analysis was completed using the NHSN statistics calculator. **Result:** Following implementation of the tiered intervention program, the CLABSI rate decreased from 1.67 to 0.62, a 62.9 percent decrease. The CLABSI SIR decreased from 1.481 to 0.553, a 62.7 percent decrease. The CLABSI SIR reduction was statistically significant (p-value of 0.0318; one tailed Z-test). **Conclusion:** Patients with coagulopathy issues pose unique challenges in infection prevention. Their abnormal clotting factors increase the risk of bleeding, making it difficult to maintain an intact and occlusive CVC dressing. Hemostatic and adhesive products are effective strategies for maintaining CVC dressing integrity and facilitating CLABSI reduction.

